# Physicochemical Properties and Cookie-Making Performance as Fat Replacer of Wax-Based Rice Bran Oil Oleogels

**DOI:** 10.3390/gels9010013

**Published:** 2022-12-26

**Authors:** Min Pang, Shengmei Kang, Lin Liu, Tengfei Ma, Zhi Zheng, Lili Cao

**Affiliations:** 1 School of Food and Bioengineering, Hefei University of Technology, Hefei 230009, China; 2 Key Laboratory for Agricultural Products Processing of Anhui Province, Hefei 230009, China; 3 Anhui Tianxiang Grain & Oil Food Co., Ltd., Fuyang 236000, China

**Keywords:** cookies, natural wax, oleogels, partial fat replacement, rice bran oil

## Abstract

Reducing the intake of trans and saturated fatty acids is a trend in healthy eating. In this study, the oleogels were prepared from rice bran oil (RBO), candle wax (CDW), beeswax (BW), rice bran wax (RBW), and carnauba wax (CRW), respectively, and the results based on their physicochemical properties and crystal structures at critical concentrations, 6 wt.%, 8 wt.%, and 10 wt.%, were determined to further investigate the oleogels as a shortening substitute in cookie recipes. Oleogel has a smooth, spreadable β′ crystal shape which creates excellent sensory properties and improves the texture, but also has some economic benefits. A comparison between the oleogels formed at critical concentrations and those with improved mass fractions was performed in several analyses such as PLM and texture, and the oleogels with higher mass fractions had a greater hardness and stickiness and denser crystal structures. This study was used to optimize the cookie recipe by partially replacing shortening with oleogel and preparing the cookies according to the 0:1, 3:7, 1:1, 7:3, 1:0 oleogel shortening mixture, respectively. Based on the results of the textural analysis, a colorimetric and sensory evaluation of the optimized formulation of oleogels in cookies, it was evident that BW and RBW oleogels have more potential to replace shortening in cookies than CDW and CRW oleogels. In particular, oleogels with a concentration of 6 wt.% RBW (RBW-6) and at a 7:3 (oleogel:shortening) shortening replacement exhibited a hardness and crispness of 15.75 N and 97.73 g, respectively, with an L* value of 66.66 and a sensory score of 22.32 ± 0.09. The value for the color perception difference (dE) between the cookies and the control group was −3.73, which allowed us to obtain a good product with a quality and characteristics similar to shortening. This supports the feasibility of new solid fats to replace traditional plastic fats in baked goods.

## 1. Introduction

Solid fats are critical to the texture and sensory properties of foods, and new plastic fats can effectively mimic traditional solid fats to bring a similar or even better quality and sensory properties to foods, so there is a growing interest in the ways to obtain new plastic fats. In the past, plastic fats for industrial applications were obtained by the partial hydrogenation of vegetable oils, which resulted in the formation of trans fatty acids [1]. In recent years, a chemical interesterification and enzymatic interesterification have become common interesterification routes. Chemical interesterification is a random rearrangement of fatty acids on the glycerol backbone using a chemical catalyst, but this route lacks specificity [2]. Compared with chemical interesterification, the enzymatic interesterification process uses specific lipase enzymes for a better specificity and control of the interesterification reaction. Although enzymatic interesterification has the advantages of mild processing conditions and has few by-products, it is costly and difficult to promote on a large scale in the food industry [3,4].

Shortening, margarine, and butter are solid fats widely used in bakeries and confectionery markets because they give products a distinctive flavor and the sensory characteristics which they need. However, these solid fats have extremely high concentrations of saturated fatty acids and some health-threatening trans fatty acids, which have been associated with an increased risk of obesity, diabetes, and cardiovascular disease [5,6,7,8]. Therefore, developing a new solid fat alternative with zero trans-fatty acids and low saturated fatty acids is essential. A gelling agent in a liquid oil is used to produce a stable three-dimensional network structure to form a supramolecular oleogel [9]. This oleogel only changes the physical state of the liquid oil; it does not affect its chemical composition [7]. The conversion of liquid oils into plasticized solids or semi-solid fats has become a trending research subject in recent years. This process can convert the harmful fatty acids generated during hydrogenation into low saturated fatty acids and zero trans-fatty acids [10], effectively reducing the risk of fatty acids to human health. The solid structure of oleogels is suitable for spreads, shortenings, and margarines and is currently used in a wide range of food products. For example, Khibani et al. applied oleogels to beef burgers [10], while Li et al. replaced shortening in cookies with monoacylglycerol and rice bran wax (RBW) oleogels to optimize cookies with a better color and crispness [11]. Alamprese applied oleogels to ice cream [12]. Sun et al. also prepared functional chocolate using oleogels [13], and Li et al. prepared oleogel-based chocolates with stable physicochemical properties and a foaming ability using different gelation mechanisms [14]. These studies prove the broad market prospect of oleogel in the food field.

Gelling agents can gel liquid oils owing to their own crystallinity, hydrophobicity, glass transition temperature, and other properties. Gelling agents can be divided into synthetic and natural gelling agents; commonly used natural gelling agents include waxes and wax esters, ethyl cellulose, fatty acids, fatty alcohols, sterol esters, lecithin, and sugar esters [15,16,17,18]. Among these, waxes are a class of lipophilic organic compounds that are highly soluble in non-polar organic solvents. Commercially valuable waxes include CDW, BW, RBW, CRW, sunflower wax, and sugarcane wax [19,20,21]. These waxes are lipids composed of esters of long-chain fatty acids and long-chain alcohols, which are in a solid state at room temperature because their melting point is between 60 °C and 86 °C. From a chemical point of view, waxes are composed of various complex compounds, mainly hydrocarbons, free fatty alcohols, free fatty acids, wax esters, sterols, and ketones. Hydrocarbons and wax esters are the main components of BW and CDW, while RBW is primarily composed of wax esters accounting for more than 90%; CRW is composed of wax esters and free fatty alcohols [22]. The gelation capacity of waxes is influenced by the percentage of these components and their chain length. Natural waxes have the property of capturing and fully integrating with liquid oil through heating and magnetic stirring to produce a stable three-dimensional mesh structure and form an oleogel system [17].

RBO is a light yellow and fragrant rice oil, which is rich in various dietary trace components and prepared from rice bran by pressing or extraction [23]. The ratio of linoleic and oleic acids in RBO is approximately 1:1.1, with different bioactive compounds and high-quality nutrients, such as phytosterols, squalene, vitamin E (α-tocopherol and tocotrienols), polyphenols, γ-glutamine, and γ-sitosterol. These trace elements impart the high bioactivity of RBO [24,25]. RBO is a healthy vegetable oil equivalent to olive oil, which is recommended by the World Health Organization as one of the top three high-value vegetable oils [26]. The main component of vitamin E in RBO is α-tocopherol, which possesses an antioxidant activity and cancer and coronary heart disease prevention effects. Moreover, RBO is abundant in γ-oryzanol, which has excellent properties such as anticancer, antioxidant, anti-cholesterol, and antidiabetic activities [24,27,28]. Hence, RBO is favored by people with cardiovascular diseases and high blood lipids. Moreover, it has a great potential in the future market of the biomass valorization field.

Therefore, in recent years, the choice of raw oil regarding the preparation of oleogels has gradually shifted from common oils to the healthier RBO [29,30,31,32,33,34]. Due to its high unsaturated fatty acid content [9], RBO, with its low viscosity and high fluidity, can form oleogels with independence, thermal reversibility, and viscoelasticity in the presence of natural waxes [35,36]. In addition, more studies on oleogels in the replacement of shortening in the preparation of cookies have focused on canola oil oleogels, corn oil oleogels, etc. [37,38,39]. However, relatively little has been reported about the application of RBO oleogels in cookies. In this study, we investigated the effects of four different natural waxes (CDW, BW, RBW, and CRW) on the gelation of RBO and explored the effects of natural waxes at different mass fractions on the physicochemical properties, thermodynamic properties and infrared spectra (in the Supplementary Materials), crystalline shape, and microstructure of oleogels. In addition, the feasibility of the partial replacement of shortening with oleogels in baked goods in terms of their texture, color, and sensory evaluation is also evaluated in this study. Thus, the present study provides a sufficient theoretical basis for applying oleogels in the food field.

## 2. Results and Discussion

### 2.1. Determination of the Critical Concentration of Oleogels (25 °C)

**Figure 1:**
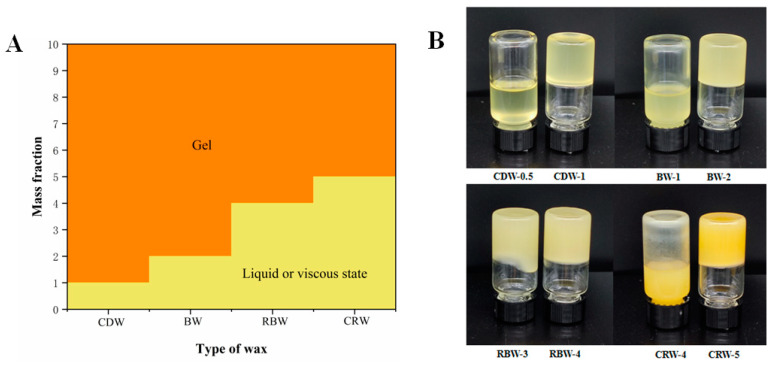
(**A**) Phase diagram of oleogels formed using natural waxes at different mass fractions (wt.%). (**B**) Critical concentration of wax-based oleogels at 25 °C.

As shown in Figure 1A,B, CDW and BW have a stronger gelling ability at room temperature, forming solid oleogels at 1 wt.% CDW (CDW-1) and 2 wt.% BW (BW-2). However, RBW and CRW have a weaker gelation ability in RBO, and an oleogel below 4 wt.% RBW (RBW-4) inverted would appear in the flowing state; CRW with the weakest gelling capacity could attain the critical state only at 5 wt.% CRW (CRW-5). The different gelling effects of the different types of waxes were mainly due to their chemical compositions. Wax esters and hydrocarbons are the main components of BW (58.00% and 26.84%, respectively) and CDW (15.76% and 72.92%, respectively). In the case of RBW, the percentage of wax esters in the components reaches more than 90%, while wax esters (62.05%) and free fatty alcohols (30.74%) were the main components of CRW. In addition, the gelling ability of the waxes may be related to the chain length of their main components, which were composed of long-chain saturated fatty acids (usually C20 to C26) and fatty alcohols (C30 to C36) [22,40]. For example, odd-length chains were the principal components in the hydrocarbons of CDW and BW, which have a stronger gelling ability. C31 accounts for more than 80% of CDW and was the most important part of the CDWs’ composition, while C27, C29, and C31 were the most significant in the BWs’ composition [22,41,42]. Considering that the state of the oleogels was closely related to the type and mass fraction (gelling agent per 100 g of oleogel) of the gelling agent, four types of oleogels will be tested in subsequent experiments at critical concentrations, 6 wt.% (wax-6), 8 wt.% (wax-8), and 10 wt.% (wax-10) mass fraction.

### 2.2. Microstructure Analysis of Oleogels (PLM)

**Figure 2:**
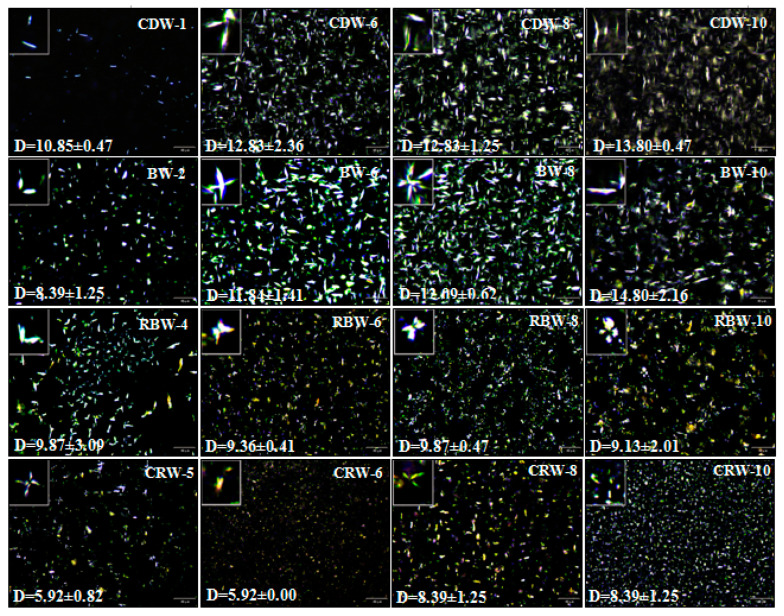
Polarized micrographs of oleogels prepared using CDW, BW, RBW, and CRW at critical concentration and 6, 8, 10 wt.% (25 °C).

Figure 2 illustrates the microstructures of natural waxes with varying gelation abilities at different mass fractions. The crystals of CDW-1 oleogels appeared as elongated rods (10.85 ± 0.47 μm), distributed evenly and irregularly. The crystal’s size significantly increases (13.32 ± 0.46 μm) with an increase in the mass fraction of CDW. As can be seen from Figure 2, the crystal distribution of BW-2 oleogels was sparse, exhibiting a stick-like structure (8.39 ± 1.25 μm). The crystal density increased significantly with an increase in the BW mass fraction; the crystal size increased to 14.80 ± 2.16 μm at BW-10, and the spatial structure was compact. The RBW-4 oleogels demonstrated a scattered and homogeneous distribution of crystals, showing a V-shaped crystal structure (9.87 ± 3.09 μm); butterfly like crystals appeared when the mass fraction of RBW increased. Although the crystal’s density increased, the increase in the crystal’s size was not significant; the spatial distribution remained scattered and uniform. RBW was a by-product of the RBO refining process, which could be dispersed in the liquid oil to form crystals and lead to liquid oil gelling [43]. In addition, CRW-5 oleogels showed a fine needle structure (5.92 ± 0.82 μm), and the length of the crystals was positively correlated with their mass fraction (Pearson coefficient: 0.89). However, the crystal’s morphology was not considerably correlated with the mass fraction, with a significant increase in the crystal’s size at CRW-8 (8.39 ± 1.25 μm) and flocculent crystallization at CRW-10 (8.39 ± 1.25 μm). The results suggest that the gelling ability [44] is influenced by the type and mass fraction of the wax and controlled by its crystal morphology. 

### 2.3. Texture Analysis of Oleogels

**Figure 3:**
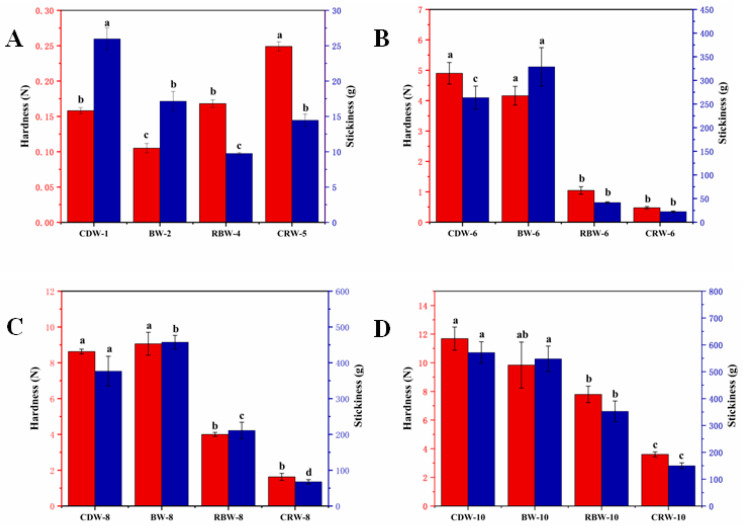
(**A**) Hardness and stickiness of CDW, BW, RBW, and CRW oleogels at critical concentrations. (**B**) Hardness and stickiness of CDW, BW, RBW, and CRW oleogels at 6 wt.%. (**C**) Hardness and stickiness of CDW, BW, RBW, and CRW oleogels at 8 wt.%. (**D**) Hardness and stickiness of CDW, BW, RBW, and CRW oleogels at 10 wt.%. The red columns represent hardness and the blue columns represent stickiness. Lower case letters represent significant differences in hardness or stickiness between different oleogels at the same concentration.

As shown in Figure 3, the hardness and stickiness values of the oleogels tended to increase with the increasing mass fraction of natural wax (*p* < 0.05). CDW oleogels exhibited the largest increasing trend, with the hardness/stickiness values increasing from 0.16 N/25.98 g at CDW-1 to 11.69 N/571.40 g at CDW-10. Under the four different mass fractions, the overall hardness/stickiness values of BW oleogels were slightly lower compared to those of CDW oleogels. From Figure 3, it can be seen that the hardness/stickiness values for the RBW and CRW oleogels were both low overall. However, according to Figure 3A, it can be seen that the hardness and stickiness of RBW-4 and CRW-5 were 0.17 N/9.76 g and 0.25 N/14.45 g, respectively, which are higher hardness and lower stickiness values than the BW-2 (0.11 N/17.14 g) and CDW-1 (0.16 N/25.98 g) oleogels at critical concentrations. The hardness/stickiness of RBW-10 and CRW-10 oleogels are 7.79 N/352.56 g and 3.61 N/150.07 g, respectively, attaining only half or even lower than that of the CDW-10 oleogels. The higher mass fraction of the natural wax resulted in the higher hardness of the corresponding oleogels [43]. In addition, Alvarez-Ramirez et al. [45] revealed that the increase in the stickiness indicated that cakes containing oleogels exhibited a greater resistance to crumbling, but that too tight a structure would harden the baked product.

### 2.4. X-ray Diffraction Analysis (XRD) of Oleogels

**Figure 4:**
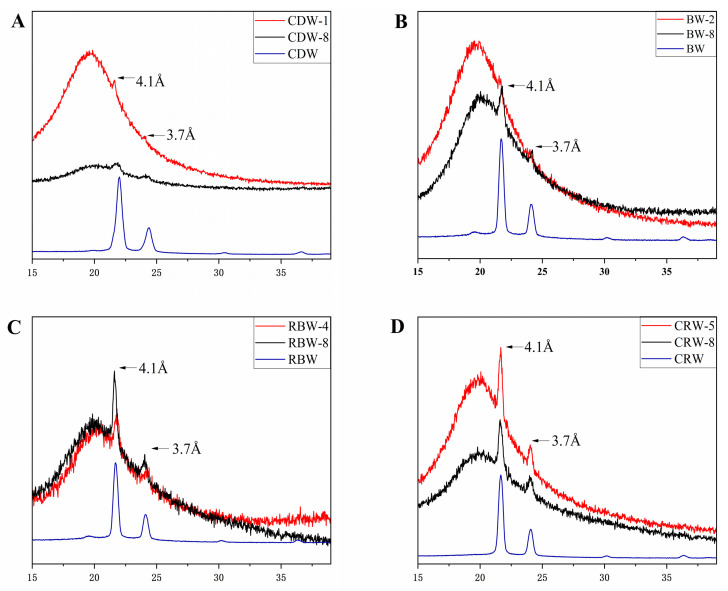
(**A**) X-ray diffraction spectra of CDW-1, CDW-8, and CDW; (**B**) X-ray diffraction spectra of BW-2, BW-8, and BW; (**C**) X-ray diffraction spectra of RBW-4, RBW-8, and RBW; (**D**) X-ray diffraction spectra of CRW-5, CRW-8, and CRW; the red line represents the critical concentration and the black line represents 8 wt.%, blue line represents gelling agent (25 °C).

The crystallinity and internal structure of the four oleogels at a critical concentration and 8 wt.% were analyzed by X-ray diffraction (XRD). Figure 4 shows the XRD patterns of the four natural waxes and their respective oleogels. From Figure 4A–D, all the oleogels exhibited peaks at 4.1 Å and 3.7 Å, belonging to the β′ crystal type. All the oleogels demonstrated strong peaks at 4.1 Å, while those at 3.7 Å exhibited weak peaks. This is because the oleogels are a semi-solid system that includes crystalline and non-crystalline zones. Among the three polycrystalline types (α, β, and β′), the β′ crystalline type is the most plastic [46]. The oleogels with a more β′ crystalline type have a cream-like, uniform, and smooth texture and belong to an orthogonal-vertical subcellular structure with a good malleability in the mouth [10]. This textural characteristic is most suitable for commercial margarine and spreads. The graph also indicated that the peak intensity increased with the increasing mass fraction of natural wax, which is identical for the four different natural waxes. This result proves that the type of gelling agent has a slight effect on the polymorphism of the wax-based oleogels. According to the previous literature [47], it was observed that the intensity of the XRD diffraction peaks of camellia oil-based oleogels samples increased with the increasing concentration of glycerol monolaurate. Combined with the XRD results of this study, it is clear that the mass fraction of the four natural waxes only affects the peak intensity of the RBO oleogels and does not change their crystalline shape.

### 2.5. Texture Analysis of Optimized Formulation of Oleogels in Cookies

**Figure 5:**
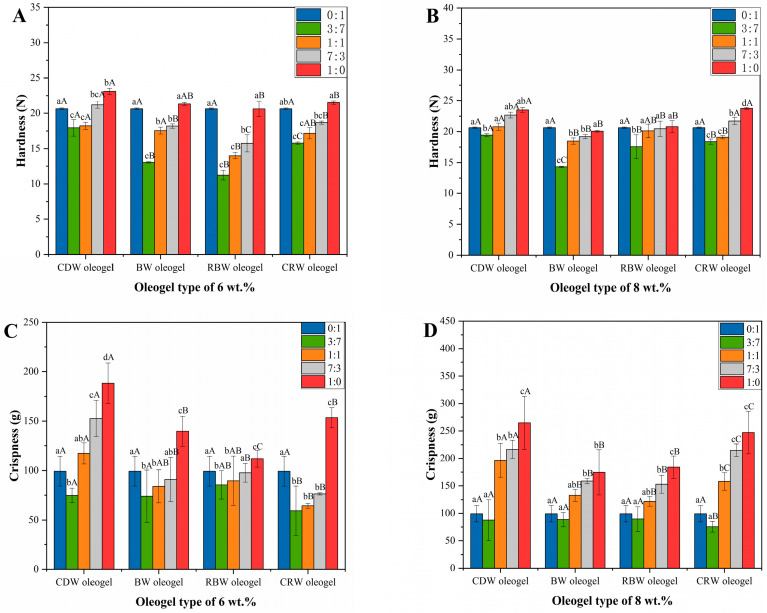
(**A**,**B**) Hardness of cookies prepared by replacing shortening with 6 and 8 wt% CDW, BW, RBW, and CRW oleogels in the ratio of 0:1, 3:7, 1:1, 7:3, 1:0 (25 °C). (**C**,**D**) Crispness of cookies prepared by replacing shortening with 6 and 8 wt.% CDW, BW, RBW, and CRW oleogels in the ratio of 0:1, 3:7, 1:1, 7:3, 1:0 (25 °C). a,b,c,d represents the significant difference in cookie hardness of the same oleogel at different addition ratios; A,B,C represents the significant difference in cookie hardness of different oleogels at the same ratio.

In this study, the oleogels at a critical concentration and 10 wt.% were not able to prepare cookies. According to the results of Section 2.3, such a phenomenon is mainly due to the lower hardness and stickiness of the critical concentration oleogels and the higher hardness and stickiness of the oleogels at 10 wt.%. Therefore, 6 wt.% and 8 wt.% oleogels were applied to cookies for the experiments. The hardness and crispness of the cookies are illustrated in Figure 5, where all four oleogels exhibited a lower hardness and crispness than shortening cookies at 6 wt.% and 8 wt.% for the 30% (3:7) shortening replacement. Because hardness is an important indicator of the ageing of baked goods, it can affect the consumer’s mouthfeel [48]. The hardness and crispness parameters for shortening cookies are 20.63 N and 99.28 g, respectively. From Figure 5A,B, the hardness of CDW oleogel cookies was the highest, and the hardness strengthened with the increase in the CDW oleogels’ mass fraction. An excessive hardness will reduce the texture of the cookies, so the CDW oleogel was not suitable as an alternative to shortening for the preparation of cookies. Although the hardness of the BW oleogel cookies was slightly higher than that of RBW oleogel cookies, the crispness was slightly lower than that of RBW oleogel cookies, as seen in Figure 5C,D. The hardness of RBW-6 oleogel cookies was lower than that of the shortening cookies (15.75 N) at 70% (7:3) shortening replacement cookies; the crispness was closest to that of the shortening cookies (97.73 g), which was the optimal ratio of the cookies substituted for shortening. The hardness increased with the increasing mass fraction of the RBW gelling agent (RBW-8), which decreases the consumer preference. CRW oleogel cookies were less crisp at CRW-6 and harder at CRW-8, making them unsuitable for the preparation of cookies. The results demonstrated that the BW and RBW oleogels were more suitable than CDW and CRW oleogels as a replacement for shortening in the preparation of cookies; the RBW-6 oleogels demonstrated the best performance at a 70% shortening replacement.

### 2.6. Color Analysis of Optimized Formulation of Oleogels in Cookies

The color parameters of the cookies were illustrated in Table 1. As can be seen from the table, the color parameters of the shortening cookies were the brightness (L* = 66.70 ± 2.86), redness (a* = 3.44 ± 1.16), and yellowness (b* = 35.40 ± 1.74). There were no particular differences between the four gelling agents in terms of the L* and b* values, but there were significant differences in the a* values (−2.89~10.30). The surface color of the cookies is darker when the L* value is lower, affecting the senses of the consumers to a certain extent, and is detrimental to cookie preparation; this is consistent with the findings of Moghtadaei et al. [49] formulated a burger using wax-based oleogels and reported a decrease in the L* values. The L* values for CDW and CRW oleogels were higher compared to BW and RBW oleogels (Table 1). However, their L* value was lower than those of BW and RBW oleogels when being used as oleogels to prepare cookies, which could be explained by the murad and caramelisation reactions that occurred during the heating of the baked products [11,50]. The cookies prepared using BW and RBW oleogels had a color closer to that of the shortening (L*, a*, and b* approximately equal to 66.00, 3.50, and 35.00, respectively), with RBW-6 oleogels having a smaller negative impact on the color parameters of the cookies than BW oleogels. In the RBW-6 oleogels, the cookies substituted with shortening according to 3:7 and 1:1 had lower a* values (0.40 ± 0.47 and 0.69 ± 0.61, respectively), while the cookies with a complete shortening substitution had lower L* values (L∗ = 61.65 ± 0.99); the cookies prepared with a 7:3 ratio of oleogel had the color parameters closest to those achieved with shortening.

### 2.7. Sensory Analysis of Optimized Formulation of Oleogels in Cookies

**Figure 6:**
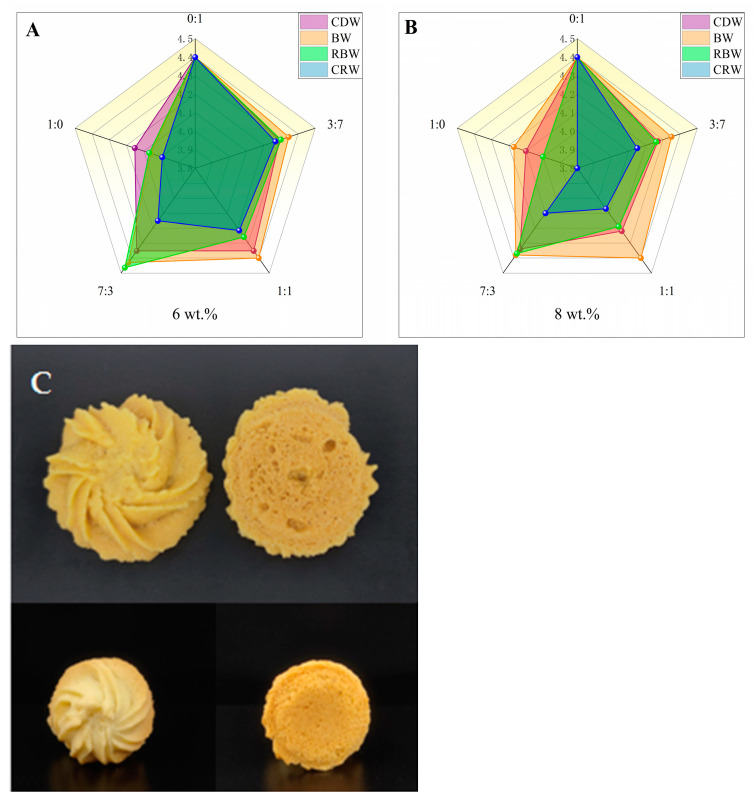
(**A**) Radar plots of the mean values of the total sensory evaluation scores of 6 wt.% CDW oleogel, BW oleogel, RBW oleogel, and CRW oleogel as shortening substitutes for the preparation of cookies at different substitution ratios were represented; (**B**) radar plots of the mean values of the total sensory evaluation scores of 8 wt.% CDW oleogel, BW oleogel, RBW oleogel, and CRW oleogel as shortening substitutes for the preparation of cookies at different substitution ratios were represented; (**C**) visual representation of shortening cookies (the picture above); visual representation of cookies with RBW-6 oleogel at 7:3 replacement shortening (the picture below).

Sixteen trained panelists were called for rating the coded cookies on a five-point scale (1—very poor, 5—very good) in five different areas. As shown in Table 2, RBW-6 oleogel added to cookies at a ratio of 7:3 instead of shortening afforded the highest score (22.32 ± 0.09). The obtained cookies were similar to the cookies obtained with shortening in terms of their odor (4.35 ± 0.12) and texture (4.44 ± 0.17) and were slightly higher in terms of their shape (4.60 ± 0.13), color (4.40 ± 0.13), and taste (4.52 ± 0.13). These results are consistent with those of the chromaticity and texture tests. Figure 6A,B represents the mean values of the total sensory evaluation for the different types of cookies; as can be seen in the graph, the sensory evaluation of the BW and RBW-8 oleogel cookies prepared with a 1:1 and 7:3 shortening substitution was equally satisfactory and they were closer to the shortening cookies. In addition, Figure 6C illustrates a visual representation of the cookies prepared with RBW-6 oleogel at a 7:3 shortening replacement and controlled with shortening cookies. Overall, RBW oleogels had the highest acceptable alternative to shortening, followed by BW oleogels, which can be used to produce baked goods that are healthier than conventional cookies. Based on previous studies, Till Wettlaufer et al. [36] concluded in the sensory evaluation of oleogel sponge cakes that although there were no significant differences in the visual appearance, odor, taste, density, crumbliness, and off-flavor among the samples, the results of significant differences in the overall impression could determine that wax-based oleogels could bring good sensory benefits to sponge cakes. Shiyi Li et al. [11] used five gelling agents to prepare different types of oleogels and applied them in the preparation of cookies, and the results of the sensory analysis showed that MAG and RBW were more acceptable in cookies.

## 3. Conclusions

In this study, the influence of the gel agent type and mass fraction on the stability and crystal structure of oleogels was investigated, and the feasibility of the application of oleogels for baking cookies was assessed. The results revealed that CDW had the strongest gelling ability and its respective oleogels as a gelling agent had the highest hardness, stickiness, and OBC. In contrast to CRW, the BW and RBW oleogels had a more cohesive crystal network. The microscopic morphology indicated that the crystal size and density of BW oleogels were positively correlated with the gelant mass fraction, whereas the size in RBW oleogels was unaffected by the gelant mass fraction. The DSC and XRD results suggest that the BW and RBW oleogels have an excellent melt temperature and smooth texture, making them suitable for an application in baked goods. The texture and color of the cookies suggests that BW and RBW are the most desirable alternatives to shortening; the BW and RBW oleogel cookies had a lower hardness and L* and crispness values closer to that of the shortening cookies. The results of the sensory evaluation showed that the cookies with 70% RBW-6 oleogel replacing shortening scored the highest. These findings provide a theoretical and practical basis for further developing foods low in saturated fatty acids and zero trans-fatty acids [38,51], driving their development in the food industry.

## 4. Materials and Methods

### 4.1. Materials and Reagents

RBO (each 100 g contains: 500 mg of γ-glutamine and 500 mg of phytosterol) was purchased from Anhui Grain World Food Ltd. (Hefei, China); food grade BW, CDW, RBW, and CRW were purchased from Changge City Yi Heng Jian apiculture Ltd. (Xuchang, China); and the base gluten flour, shortening, powdered sugar, eggs, and baking powder were all purchased from local markets (Hefei, China).

### 4.2. Preparation of Oleogels

A total of 30 g of RBO were weighted in 50 mL beakers at room temperature, and four different natural waxes were weighed with mass fractions of 1 wt.%, 2 wt.%, 3 wt.%, 4 wt.%, 5 wt.%, 6 wt.%, 7 wt.%, 8 wt.%, 9 wt.%, and 10 wt.%, respectively. We placed the accurately weighed material in a beaker, added the magnetic rotor, and sealed with cling film. Then, they were placed in a thermostatic water bath heated to 90 °C and stirred for 10 min under the action of a magnetic stirrer. We mixed until the liquid oil was clarified and there was no visible particulate matter and set them aside at room temperature for 24 h.

### 4.3. Preparation of Oleogel Application in Cookies

According to the methodology of Shiyi Li et al. for cookies [11], the recipe for optimized cookies was determined with some modifications. The ingredients of the cookies included 50 g of base gluten flour, 30 g of oil, 15 g of powdered sugar, 10 g of egg, and 1 g of baking powder. The total 30 g of oil were set as the oleogel: the shortening ratios were at 0:1, 3:7, 1:1, 7:3, and 1:0, respectively. We added powdered sugar to the weighed oil and mixed well. Then, we added eggs in small amounts several times and beat until white. Baking powder and low gluten flour were added and the mixture was stir and beat. They were then baked in a preheated oven at 180 °C on top and 160 °C on bottom for 20 min. We made 6 cookies per group and measured their size after they had been cooling for 2 h.

### 4.4. Determination of the Critical Concentration of Oleogels 

The oleogel samples of different mass fractions prepared from four different natural waxes were poured into 10 mL glass vials and left for 24 h at room temperature, then all samples were inverted for 10 min. The critical concentration was the concentration of the oleogel samples prepared from the minimum mass fraction of natural waxes that did not flow when inverted.

### 4.5. Polarized Light Microscopy (PLM) of Oleogels 

We aspirated 20 μm drops of melted oleogel onto pre-warmed slides and quickly covered with coverslips to form a uniformly distributed oleogel sample; this was cooled at room temperature and left for 24 h. Under the microscope, the crystal structure was observed with a 40× objective. The microstructure of the oleogels was characterized by an MP 30 polarized light microscope (Mshot, Guangzhou, China) with a camera attached. All PLM photos were taken at 40× magnification.

### 4.6. Texture Analysis of Oleogels

The TA-XT plus Texture Analyzer (Stable Microsystems, Surrey, UK) was used to determine the hardness and stickiness of the oleogel samples. A P-0.5 probe with a diameter of 1.25 cm and a length of 4 cm was inserted into the sample at a probing speed of 1 mm/s, withdrawn from the sample at a speed of 10 mm/s after reaching a maximum probing depth of 10 mm, and set to a return height of 40 mm. The hardness and stickiness of the samples were calculated using Texture Exponent v.6.1.16.0 software (Stable Microsystems) and each test was repeated three times.

### 4.7. X-ray Diffraction Analysis (XRD) of Oleogels

The crystalline structure of the oleogel samples was determined by an X-ray diffractometer (PANalytical B.V., Almelo, the Netherlands) equipped with a copper X-ray tube (λ = 1.54 Å) at 40 kV and 40 mA. The diffraction patterns were measured by a scintillation detector with scanning angles of 5° to 50° (2θ). The X-ray diffraction patterns of the samples were analyzed using MDI Jade 6.0 software (Materials Data Ltd., Livermore, CA, USA).

### 4.8. Texture Analysis of the Optimized Formulation of Oleogels in Cookies

The hardness and crispness of the cookies with similar specifications were tested by a TA-XT Plus physical property tester (Stable Microsystems, Surrey, UK). The HDP/BS probe was selected to press down on the cookie at a test speed of 2.00 mm/s, reach a depth (10 mm) sufficient to crush the cookie, and then return at 10 mm/s. The maximum peak force required to crush the cookies was recorded as the hardness of the cookies. The determination of the crispness was at the point with its first significant peak during the compression of the probe into the cookie. The hardness and crispness of the cookies were calculated using Texture Exponent v.6.1.16.0 software (Stable Microsystems) and each test was repeated three times. All tests were performed at room temperature (25 °C).

### 4.9. Color Analysis of the Optimized Formulation of Oleogels in Cookies

The average width and thickness of the cookies was measured according to Areum Jang from the AACC-approved method (10-52, AACC (2009)). The color parameters were measured with a spectrophotometer: lightness (L*: ± bright/dark); redness (a*: ± red/green); and yellowness (b*: ± yellow/blue), ΔE* represents the total color difference [39,52]. The parameters of the standard plate are L* = 94.10; a* = −0.15; and b* = 4.55. The actual L*, a*, and b* values of the standard plate are compared with the values measured by the software and the calibration curves of the color parameters are plotted, and each test is repeated three times.

### 4.10. Sensory Analysis of the Optimized Formulation of Oleogels in Cookies

The sensory analysis was performed by 16 trained panel members (8 males and 8 females, aged 18–35 years) who rated the coded cookie samples on a test scale (1—very poor, 5—very good) according to their preferences. According to GB/T 20980-2007 and GB 7100–2015, the cookies were evaluated on five components: their shape; color; smell; texture; and taste. The cookies were coded and randomly assigned to panel members, each of whom cleaned their mouths with mineral water between assessing the different cookies.

### 4.11. Statistical Analysis

Microsoft office excel 2007 and Origin 9.0 (OriginLab, Northampton, MA, USA) were used for the data processing and graphing. The Pearson correlation analysis and Analysis of variance (ANOVA) with Tukey’s test were performed using SPSS 18.0 statistical software (SPSS Inc, Chicago, IL, USA) with a significance threshold of 5% (*p* < 0.05). The data obtained in this experiment were all the average values of the three repeated experiments, and the data results were expressed as the average ± standard deviation.

## Figures and Tables

**Table 1:** The color parameters of cookies prepared using CDW, BW, RBW, and CRW oleogels at 6 wt.% and 8 wt.% in different proportions of shortening replacement (25 °C). a,b,c,d represents the significant difference in color of different samples.

Sample	L*	a*	b*	ΔE*	Sample	L*	a*	b*	ΔE*
Control	66.70 ± 2.86 ^ab^	3.44 ± 1.16 ^b^	35.40 ± 1.74 ^b^	41.51 ± 1.89 ^b^					
CDW	72.59 ± 2.13 ^c^	−2.90 ± 0.15 ^c^	34.37 ± 0.99 ^b^	37.96 ± 0.21 ^a^	BW	60.14 ± 1.23 ^b^	−2.70 ± 0.18 ^c^	47.77 ± 1.12 ^a^	44.77 ± 1.30 ^b^
CDW-6					BW-6				
3:7	67.13 ± 1.01 ^a^	0.24 ± 0.38 ^a^	32.45 ± 0.75 ^ab^	36.65 ± 0.30 ^a^	3:7	66.56 ± 0.92 ^ab^	2.90 ± 0.95 ^b^	32.34 ± 0.47 ^b^	38.73 ± 0.69 ^a^
1:1	66.94 ± 1.39 ^a^	0.24 ± 0.44 ^a^	30.40 ± 1.21 ^a^	36.49 ± 1.33 ^a^	1:1	66.20 ± 1.15 ^ab^	2.74 ± 1.10 ^b^	31.57 ± 1.52 ^b^	38.11 ± 1.24 ^a^
7:3	64.09 ± 1.38 ^b^	1.99 ± 0.36 ^ab^	30.20 ± 1.33 ^a^	40.36 ± 1.31 ^ab^	7:3	65.77 ± 1.03 ^a^	3.49 ± 1.29 ^a^	30.25 ± 2.57 ^b^	37.75 ± 1.46 ^a^
1:0	63.37 ± 1.66 ^b^	2.23 ± 0.73 ^ab^	28.93 ± 1.01 ^c^	40.11 ± 1.02 ^ab^	1:0	64.50 ± 1.70 ^a^	4.52 ± 0.17 ^a^	29.13 ± 2.62 ^c^	40.79 ± 0.98 ^ab^
CDW-8					BW-8				
3:7	65.42 ± 1.18 ^ab^	1.54 ± 0.92 ^a^	30.47 ± 0.52 ^a^	37.63 ± 1.02 ^a^	3:7	65.20 ± 0.45 ^a^	3.04 ± 0.51 ^b^	33.42 ± 0.35 ^a^	40.96 ± 0.61 ^ab^
1:1	64.03 ± 0.32 ^b^	1.88 ± 1.33 ^ab^	29.33 ± 0.82 ^a^	36.85 ± 0.43 ^c^	1:1	65.13 ± 0.86 ^a^	2.56 ± 0.77 ^a^	32.39 ± 0.64 ^a^	38.85 ± 0.44 ^a^
7:3	63.12 ± 1.06 ^b^	2.46 ± 0.75 ^b^	28.46 ± 1.36 ^c^	39.65 ± 0.46 ^a^	7:3	64.70 ± 1.48 ^a^	2.35 ± 0.65 ^a^	30.15 ± 1.05 ^b^	39.07 ± 0.72 ^a^
1:0	62.57 ± 0.48 ^b^	2.56 ± 0.84 ^b^	27.85 ± 1.03 ^d^	40.06 ± 0.33 ^ab^	1:0	64.15 ± 1.38 ^b^	1.11 ± 0.33 ^c^	29.67 ± 0.93 ^c^	35.53 ± 0.49 ^c^
RBW	76.47 ± 2.49 ^c^	−2.49 ± 0.49 ^c^	36.80 ± 2.34 ^b^	36.91 ± 0.96 ^a^	CRW	62.49 ± 0.54 ^b^	1.30 ± 0.37 ^c^	42.92 ± 0.74 ^a^	54.02 ± 0.68 ^d^
RBW-6					CRW-6				
3:7	70.40 ± 0.32 ^a^	3.40 ± 0.47 ^a^	33.85 ± 1.23 ^ab^	37.40 ± 0.88 ^a^	3:7	64.81 ± 1.89 ^a^	2.46 ± 1.00 ^a^	31.35 ± 2.38 ^b^	40.55 ± 0.73 ^ab^
1:1	67.44 ± 1.52 ^ab^	2.69 ± 0.61 ^a^	29.16 ± 0.28 ^c^	36.16 ± 1.13 ^c^	1:1	64.21 ± 0.69 ^a^	1.01 ± 0.61 ^c^	30.41 ± 1.20 ^a^	37.30 ± 1.33 ^a^
7:3	66.66 ± 2.81 ^ab^	2.08 ± 0.55 ^ab^	28.39 ± 2.35 ^c^	37.78 ± 1.10 ^a^	7:3	64.11 ± 1.16 ^a^	1.34 ± 0.17 ^c^	28.56 ± 0.69 ^c^	39.71 ± 0.51 ^a^
1:0	64.65 ± 0.99 ^b^	1.40 ± 0.07 ^b^	27.15 ± 0.53 ^d^	38.40 ± 0.57 ^a^	1:0	62.95 ± 0.80 ^b^	2.24 ± 1.04 ^b^	27.28 ± 1.73 ^c^	39.87 ± 0.54 ^a^
RBW-8					CRW-8				
3:7	69.90 ± 2.49 ^a^	3.08 ± 0.17 ^a^	31.41 ± 0.40 ^a^	36.14 ± 1.38 ^a^	3:7	64.28 ± 0.82 ^a^	1.43 ± 0.27 ^c^	28.98 ± 1.56 ^c^	39.65 ± 0.43 ^a^
1:1	67.18 ± 2.15 ^ab^	3.22 ± 0.24 ^a^	29.60 ± 0.85 ^a^	34.89 ± 1.36 ^c^	1:1	63.84 ± 0.76 ^b^	2.35 ± 0.76 ^b^	28.23 ± 1.21 ^c^	37.63 ± 0.82 ^a^
7:3	65.00 ± 3.40 ^b^	2.43 ± 0.19 ^ab^	28.56 ± 0.88 ^c^	38.15 ± 1.56 ^a^	7:3	62.45 ± 1.12 ^b^	2.44 ± 0.63 ^b^	27.65 ± 0.43 ^c^	40.10 ± 0.12 ^ab^
1:0	64.57 ± 0.18 ^b^	1.16 ± 0.22 ^b^	26.23 ± 1.82 ^d^	37.26 ± 0.44 ^a^	1:0	62.02 ± 0.74 ^c^	2.65 ± 1.48 ^ab^	26.59 ± 0.78 ^d^	41.82 ± 0.38 ^c^

**Table 2:** Sensory parameters of cookies prepared using CDW, BW, RBW, and CRW oleogels at critical concentration and 8 wt.% in different proportions of shortening replacement after evaluation by 20 evaluators (25 °C). a,b,c represents the significant difference in sensory evaluation of different samples.

Sample	Shape (5′)	Color (5′)	Odor (5′)	Texture (5′)	Tatse (5′)	Total (5′)
0:1	4.51 ± 0.18 ^a^	4.34 ± 0.12 ^a^	4.32 ± 0.32 ^a^	4.40 ± 0.29 ^a^	4.45 ± 0.33 ^a^	22.02 ± 0.07 ^a^
CDW-6						
3:7	4.40 ± 0.13 ^a^	4.28 ± 0.12 ^a^	4.32 ± 0.16 ^a^	4.20 ± 0.17 ^a^	4.26 ± 0.16 ^a^	21.46 ± 0.07 ^a^
1:1	4.44 ± 0.17 ^a^	4.32 ± 0.16 ^a^	4.30 ± 0.14 ^a^	4.34 ± 0.24 ^a^	4.36 ± 0.17 ^a^	21.76 ± 0.05 ^a^
7:3	4.46 ± 0.08 ^ab^	4.26 ± 0.21 ^a^	4.30 ± 0.19 ^a^	4.32 ± 0.16 ^a^	4.42 ± 0.17 ^ab^	21.76 ± 0.08 ^a^
1:0	4.36 ± 0.12 ^a^	4.30 ± 0.19 ^a^	4.12 ± 0.25 ^a^	3.96 ± 0.19 ^a^	4.02 ± 0.16 ^a^	20.76 ± 0.16 ^a^
CDW-8						
3:7	4.36 ± 0.17 ^a^	4.32 ± 0.21 ^a^	4.24 ± 0.16 ^a^	4.20 ± 0.19 ^ab^	4.24 ± 0.16 ^a^	21.36 ± 0.06 ^ab^
1:1	4.24 ± 0.16 ^ab^	4.30 ± 0.14 ^a^	4.14 ± 0.12 ^a^	4.24 ± 0.16 ^ab^	4.20 ± 0.19 ^ab^	21.12 ± 0.05 ^b^
7:3	4.48 ± 0.16 ^a^	4.36 ± 0.19 ^a^	4.26 ± 0.14 ^a^	4.30 ± 0.19 ^a^	4.30 ± 0.19 ^a^	21.7 ± 0.08 ^a^
1:0	4.04 ± 0.21 ^ab^	4.32 ± 0.16 ^a^	4.10 ± 0.13 ^ab^	4.04 ± 0.15 ^a^	3.98 ± 0.13 ^a^	20.48 ± 0.12 ^ab^
BW-6						
3:7	4.28 ± 0.19 ^a^	4.40 ± 0.12 ^a^	4.30 ± 0.10 ^a^	4.40 ± 0.12 ^a^	4.35 ± 0.12 ^a^	21.73 ± 0.05 ^a^
1:1	4.40 ± 0.12 ^a^	4.44 ± 0.21 ^a^	4.32 ± 0.25 ^a^	4.42 ± 0.29 ^a^	4.42 ± 0.22 ^a^	22.00 ± 0.04 ^a^
7:3	4.58 ± 0.10 ^a^	4.30 ± 0.19 ^a^	4.32 ± 0.10 ^a^	4.40 ± 0.25 ^a^	4.55 ± 0.19 ^a^	22.15 ± 0.12 ^a^
1:0	4.08 ± 0.28 ^ab^	4.16 ± 0.21 ^a^	4.00 ± 0.00 ^a^	4.04 ± 0.30 ^a^	4.02 ± 0.26 ^a^	20.30 ± 0.06 ^a^
BW-8						
3:7	4.36 ± 0.12 ^a^	4.26 ± 0.16 ^a^	4.32 ± 0.18 ^a^	4.42 ± 0.12 ^a^	4.40 ± 0.24 ^a^	21.76 ± 0.06 ^a^
1:1	4.50 ± 0.19 ^a^	4.36 ± 0.12 ^a^	4.32 ± 0.12 ^a^	4.40 ± 0.09 ^a^	4.48 ± 0.35 ^a^	22.06 ± 0.07 ^a^
7:3	4.46 ± 0.21 ^a^	4.34 ± 0.24 ^a^	4.26 ± 0.22 ^a^	4.36 ± 0.20 ^a^	4.46 ± 0.15 ^a^	21.88 ± 0.08 ^a^
1:0	4.30 ± 0.19 ^a^	4.30 ± 0.19 ^a^	4.16 ± 0.14 ^a^	4.10 ± 0.19 ^a^	4.00 ± 0.06 ^a^	20.86 ± 0.12 ^a^
RBW-6						
3:7	4.40 ± 0.13 ^a^	4.26 ± 0.16 ^a^	4.28 ± 0.20 ^a^	4.30 ± 0.11 ^a^	4.26 ± 0.16 ^a^	21.50 ± 0.05 ^a^
1:1	4.36 ± 0.10 ^a^	4.28 ± 0.17 ^a^	4.26 ± 0.16 ^a^	4.06 ± 0.22 ^a^	4.34 ± 0.10 ^a^	21.30 ± 0.11 ^a^
7:3	4.60 ± 0.13 ^a^	4.40 ± 0.13 ^a^	4.36 ± 0.12 ^a^	4.44 ± 0.17 ^a^	4.52 ± 0.13 ^a^	22.32 ± 0.09 ^a^
1:0	4.08 ± 0.19 ^ab^	4.18 ± 0.12 ^a^	4.02 ± 0.12 ^a^	4.02 ± 0.12 ^a^	4.04 ± 0.10 ^a^	20.34 ± 0.06 ^a^
RBW-8						
3:7	4.34 ± 0.10 ^a^	4.14 ± 0.22 ^a^	4.26 ± 0.14 ^a^	4.26 ± 0.16 ^ab^	4.32 ± 0.16 ^a^	21.32 ± 0.07 ^ab^
1:1	4.10 ± 0.13 ^b^	4.32 ± 0.16 ^a^	4.26 ± 0.16 ^a^	4.06 ± 0.12 ^b^	4.22 ± 0.20 ^ab^	20.96 ± 0.10 ^ab^
7:3	4.54 ± 0.21 ^a^	4.36 ± 0.12 ^a^	4.26 ± 0.16 ^a^	4.40 ± 0.18 ^a^	4.30 ± 0.19 ^a^	21.86 ± 0.10 ^a^
1:0	4.06 ± 0.08 ^ab^	4.16 ± 0.14 ^a^	4.02 ± 0.17 ^ab^	4.04 ± 0.10 ^a^	3.88 ± 0.12 ^a^	20.16 ± 0.09 ^b^
CRW-6						
3:7	4.32 ± 0.19 ^a^	4.28 ± 0.23 ^a^	4.28 ± 0.15 ^a^	4.22 ± 0.23 ^a^	4.24 ± 0.19 ^a^	21.34 ± 0.03 ^a^
1:1	4.26 ± 0.21 ^a^	4.26 ± 0.21 ^a^	4.22 ± 0.23 ^a^	4.12 ± 0.16 ^a^	4.22 ± 0.17 ^a^	21.08 ± 0.05 ^a^
7:3	4.22 ± 0.17 ^b^	4.24 ± 0.19 ^a^	4.06 ± 0.22 ^a^	4.14 ± 0.08 ^a^	4.10 ± 0.20 ^b^	20.76 ± 0.07 ^bc^
1:0	3.96 ± 0.16 ^b^	4.14 ± 0.15 ^a^	3.94 ± 0.17 ^a^	3.86 ± 0.14 ^a^	4.06 ± 0.08 ^a^	19.96 ± 0.10 ^a^
CRW-8						
3:7	4.20 ± 0.17 ^a^	4.18 ± 0.13 ^a^	4.16 ± 0.10 ^a^	4.06 ± 0.08 ^b^	4.16 ± 0.19 ^a^	20.76 ± 0.05 ^b^
1:1	4.14 ± 0.15 ^b^	4.18 ± 0.13 ^a^	4.10 ± 0.13 ^a^	3.98 ± 0.16 ^b^	3.94 ± 0.14 ^b^	20.34 ± 0.09 ^c^
7:3	4.16 ± 0.19 ^a^	4.14 ± 0.15 ^a^	4.18 ± 0.13 ^a^	3.90 ± 0.13 ^b^	4.14 ± 0.15 ^a^	20.52 ± 0.10 ^b^
1:0	3.82 ± 0.24 ^b^	4.18 ± 0.13 ^a^	3.78 ± 0.25 ^b^	3.76 ± 0.30 ^a^	3.80 ± 0.28 ^a^	19.34 ± 0.16 ^c^

## Data Availability

The data that support the findings of this study are available upon request by contact with the corresponding author.

## Supplementary Materials

The following are available online at https://www.mdpi.com/article/10.3390/gels9010013/s1, Figure S1: Thermograms of wax-based oleogels, Figure S2: Infrared spectra of oleogels, Table S1: Oil binding capacity of CDW, BW, RBW, and CRW oleogels, Table S2: Thermodynamic parameters of the crystallization and melting processes of oleogels. References [9,48,53,54,55] are cited in the Supplementary Materials.
