# Supplementary material for: Physicochemical Properties and Cookie-Making Performance as Fat Replacer of Wax-Based Rice Bran Oil Oleogels
Source: Gels. 2022 Dec 26;9(1):13. doi: 10.3390/gels9010013 (PMC9858516; doi:10.3390/gels9010013)
Supplement: Supplementary file 1 [file gels-09-00013-s001.zip › gels-2080021-supplementary.pdf]

Supplementary Materials

Physicochemical properties and cookie-making performance as fat replacer of wax-based rice bran oil oleogels

Min Pang[a b], Shengmei Kang[a b], Lin Liu[a b], Tengfei Ma[c], Zhi Zheng[a b], Lili Cao[a b*]

[a]*School of Food and Bioengineering, Hefei University of Technology, Hefei, 230009, P. R. China*

[b]*Key Laboratory for Agricultural Products Processing of Anhui Province, Hefei, 230009, P. R. China*

[c]*Anhui Tianxiang Grain&Oil Food Co. LTD, Fuyang, 236000，P. R. China*

Running title: Characterization and application of rice bran oil oleogel

**Corresponding author:*

Lili Cao, lilycao504@hfut.edu.cn

SI. Methods

SI-1. Oil binding capacity (OBC)

Referring to the method of *Mustafa Ög˘ütcü* [1] with some modification, appropriate amounts of 1.5 mL centrifuge tubes were taken, marked and weighed, and record the weight a.Pipette precisely aspirate 1 mL of oleogel in a centrifuge tube, leave it at room temperature for 24 h and then weigh it and record the weight b. All samples were placed symmetrically in a high-speed centrifuge and centrifuged at 10,000 rpm for 15 min. After centrifugation, open the cap and invert on filter paper to remove the centrifuged liquid oil, invert for 3 min, then weigh and record the weight c.

$$\% \text{ Released oil} = [(b - a) - (c - a)]/(b - a) \times 100$$

$$\% \text{ OBC} = 1 - \% \text{ Released oil}$$

SI-2. Differential scanning calorimetry (DSC)

The thermal properties of the oleogel samples were studied relative to an empty aluminum pan used as a reference using a Q2000 differential scanning calorimetry (TA Instruments, New Castle, DE, USA). Accurately weight 7~8 mg sample and seal in aluminum pan.At room temperature, it was heated to 100°C at 20°C/min while holding for 5 min to eliminate the crystallization memory; then cooled to 0°C at 10°C/min while holding for 5 min to

completely crystallize; and finally heated to 100°C at 10°C/min. Calculation of parameters in thermal behaviour by TA Universal Analysis software.

SI -3. Fourier transform infrared spectrometry (FT-IR) analysis

Possible structural interactions in oleogel samples were analyzed using a Nicolet 67 Fourier transform infrared spectrometer equipped with a Smart iTX ATR sampling accessory. The instrument operated in attenuated total reflection mode (ATR) and the spectra were acquired in the wavenumber range of 4000-550 cm^{-1} with 32 scans.

SII. Results and Discussion

S Ⅱ-1. Oil binding capacity (OBC)

The OBC of an oleogel is the oil holding capacity of the gelling agent. The strength of the OBC represents the ability of the crystalline network structure to capture and hold the liquid oil, which corresponds to the ability of the liquid oil to attach to a system without flowing out. OBC plays a critical role in assessing the potential of oleogels in food applications and is an important indicator for measuring the stability of oleogels. As shown in **Table S1**, the CLW and BW oleogels exhibited a strong OBC value of 99.57 % and 99.17 %, respectively, at the critical concentration; the OBC of RBW and CRW oleogels were 62.84 % and 56.42 %, respectively. The OBC of BW and CLW oleogels was 100 % at 6 and 8 wt.%, with no precipitation of liquid oil. Although RBW

and CRW oleogels demonstrated liquid oil precipitation, their OBC values were 86.70 ± 2.17 % and 64.78 ± 1.57 %, respectively, with CRW oleogels exhibiting poor oil holding properties. No liquid oil precipitated out of the four types of oleogels at 10 wt.%, which indicates that the network structure in the oleogel system is more stable when the concentration of the gelling agent reaches a specific value. The results showed that the OBC of the oleogels was closely related to the type and mass fraction of the gelling agent. BW and CLW oleogels had the best OBC, while RBW and CRW oleogels had poor OBC; CLW oleogels had the strongest OBC while that of CRW oleogels was the weakest. According to previous studies, the OBC of oleogels depends on the crystallization ability of the different gelling agents [2]. The OBC of the oleogels significantly increased with an increase in the gelant mass fraction ($P < 0.05$). OBC results can also be explained by the crystal structure observed in polarized light microscopy (PLM), OBC is derived from the uniform and dense network structure formed between the crystals in the oleogel system. When the mass fraction of gelata increases, the crystal network in the oleogel system is compact, which can efficiently seal the liquid oil in the system and enhance OBC.

SII-2. Thermal properties of oleogels

The melting and crystallization thermal behavior of oleogels are vital physicochemical properties that affect their macroscopic behavior. **Fig. S1**

shows the DSC thermograms of the four natural waxes and their respective oleogel samples. The melting and crystallization parameters are listed in **Table S2**. The different thermal profiles of the natural waxes can be attributed to their varying compositions. Two melting peaks and one crystallization peak were observed for all four different mass fractions of BW oleogels. The temperature corresponding to the melting and crystallization peaks increased with increasing BW mass fraction; however, a significant difference was observed between the melting and crystallization peaks ($p < 0.05$). The temperature of the melting peak temperature was higher than that of the crystallization peak because wax esters (58.00 %) and hydrocarbons (26.84 %) were the main chemical components of BW. The melting and crystallization peaks were not detected for the CLW oleogels at the critical concentration (in combination with the critical concentration results) due to the low mass fraction of CLW (1 wt.%). This phenomenon resulted in the formation of less number of crystals in the oleogels. The melting and crystallization processes were brief for detection, which is consistent with the results for CLW oleogels in the PLM. Moreover, the melting and crystallization peaks of CLW were considerably broad. Thus, it is difficult to observe the crystalline phase change process at lower mass fractions of natural wax. With the increasing mass fraction of CLW, only one melting and one crystallization peak was observed for CLW oleogels. The temperature of these peaks increased owing to hydrocarbons (72.92 %), which are the main component of CLW. The RBW

oleogel has two melting peaks and one crystallization peak at different mass fractions of the oleogels. The oleogels of BW and RBW have the same number of peaks during melting and crystallization. However, the temperatures of the melting and crystallization peaks of the BW oleogels were lower than those corresponding to the RBW oleogels due to the influence of the wax ester content of the RBW component on the melting point. The crystallization and melting process of CRW oleogels is complicated by the presence of two peaks during both melting and crystallization, a phenomenon that can be explained by the composition of CRW. In addition, the crystallization and melting peaks of CRW oleogels corresponded to high temperatures and reached a complete melting temperature of approximately 81 °C, possibly producing a distinct waxy texture in the mouth when applied to baked goods. Overall, the temperatures of the melting and crystallization peaks of the BW and CLW oleogels were lower than those corresponding to the RBW and CRW oleogels at different mass fractions. Furthermore, Davidovich-Pinhas et al. [3] demonstrated that liquid oils did not exhibit thermal behavior, indicating that the change of enthalpy was only influenced by the type and mass fraction of the natural waxs. Additionally, they suggested that the enthalpy change of the oleogels significantly increased with increasing mass fraction of the natural wax ($p < 0.05$), implying that the gel system had a homogeneous and ordered arrangement of crystal molecules and a high degree of spontaneity in converting liquid oil into semi-solid fats.

SII-3. Fourier transform infrared spectrometry (FT-IR) analysis

The interactions of the chemical bonds in the gel system can be obtained from the infrared spectrograms, thus determining the structural information of the gel molecules. **Fig. S2** illustrates the infrared spectrograms of the four oleogel samples at critical concentration and 8 wt.%, which are compared with liquid oils and natural waxes. As shown in **Fig. S2**, the oleogels have similar absorption peaks at different mass fractions, representing the location of the absorption peaks independent of the gelling agent concentration. The four oleogels have defined absorption peaks at 3009, 2923, 2852, 1743, 1462, 1376, 1240, 1160, 1095, and 722 cm^{-1}. The absorption peak at 722 cm^{-1} represents more than four CH_2 linearly connected planar oscillations. The absorption peak at 1095 cm^{-1} corresponds to the stretching vibration of the saturated aliphatic ether (C-O-C); absorption peaks at 1160 cm^{-1} and 1240 cm^{-1} may be related to the C-O stretching of C-O-C and C-O-H. The absorption peak at 1376 cm^{-1} is ascribed to the symmetric deformation vibration of CH_3 while that at 1462 cm^{-1} is associated with the deformation and antisymmetric vibrations of $-CH_2$ and $-CH_3$, respectively. The absorption peak at 1743 cm^{-1} is related to the ester (C=O) carbonyl stretching vibration, while those at 2852 cm^{-1} and 2923 cm^{-1} correspond to the C-H stretching vibrations in the saturated hydrocarbon CH_2. The absorption peak at 3009 cm^{-1} is ascribed to the C-H stretching vibration of olefins and aromatics. A review of the

literature shows that intramolecular and intermolecular hydrogen bonding is responsible for the semi-solid state of the oleogel [4]. However, no hydrogen bonding was found in the infrared spectrograms of the four oleogel samples, which may be related to the van der Waals forces, hydrophobicity, and self-assembly mechanisms of the gel system [4,5].

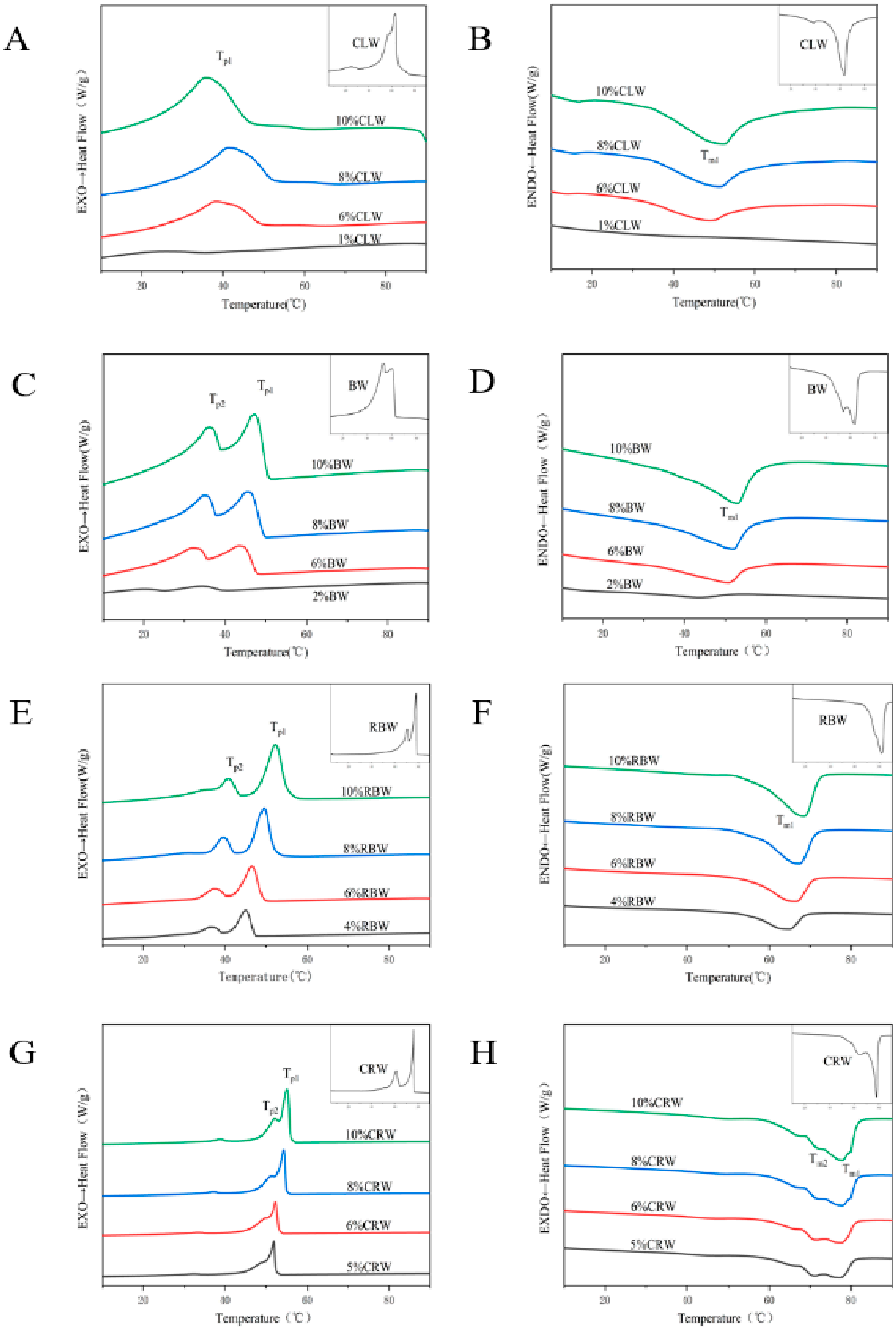

Fig.S1. Thermograms of wax-based oleogels at critical concentration and 6, 8, 10 wt.%. (A, B) Crystallization and melting curves for CLW and CLW oleogels, (C, D) crystallization and melting curves for BW and BW oleogels, (E, F) crystallization and melting curves for RBW and CRW oleogels (G, H) crystallization and melting curves for CRW and CRW oleogels. The upper right-hand corner of the graph shows the crystallisation and melting curves corresponding to natural waxes.

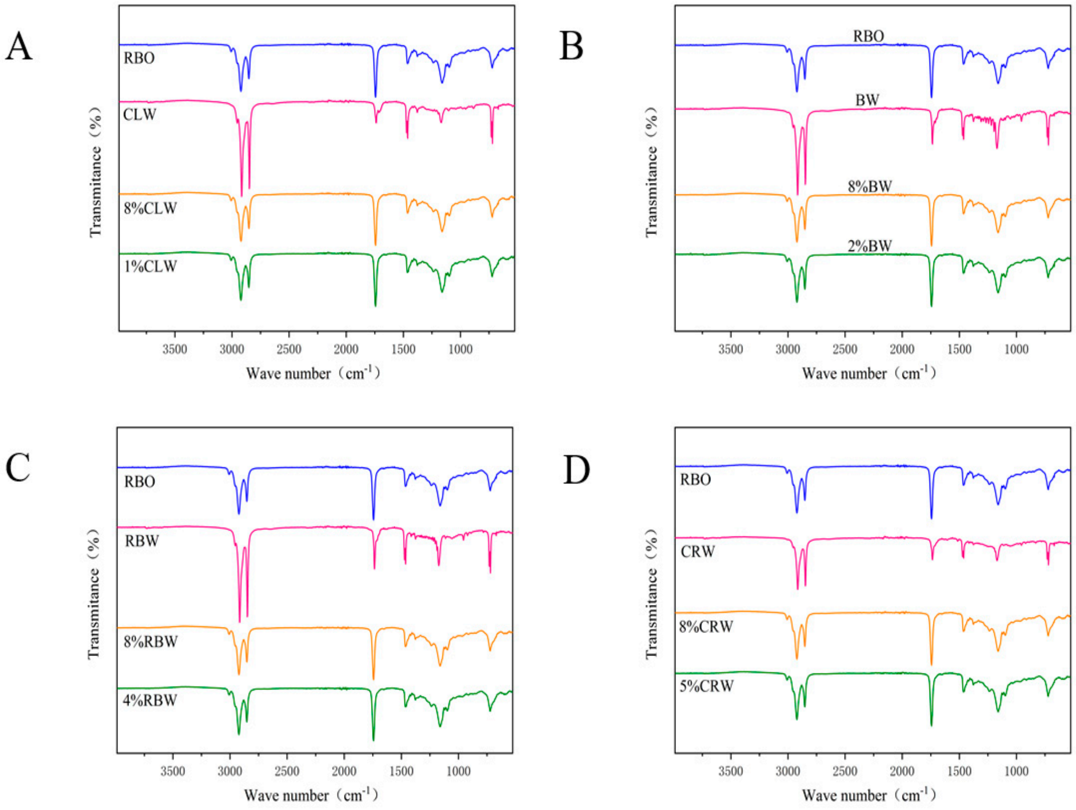

Fig.S2. Infrared spectra of RBO, gelling agents, oleogels at critical concentration and 8 wt.% (25 °C).

Table.S1. OBC of CLW, BW, RBW, and CRW oleogels at critical concentration, 6, 8, and 10 wt.% at 10,000 rpm (25 °C).

**Samples**	**OBC（%）**			
	**Critical concentration**	**6 wt.%**	**8 wt.%**	**10 wt.%**
CLW oleogels	99.57±0.61^{a}(1 wt.%)	100±0.00^{a}	100±0.00^{a}	100±0.00^{a}
BW oleogels	99.17±0.58^{a} (2 wt.%)	100±0.00^{a}	100±0.00^{a}	100±0.00^{a}
RBW oleogels	62.84±1.60^{c} (4 wt.%)	86.70±2.17^{b}	96.99±1.57^{a}	100±0.00^{a}
CRW oleogels	56.42±1.20^{d} (5 wt.%)	64.78±1.57^{c}	85.86±1.05^{b}	100±0.00^{a}

Values in the same row with different superscript letters are significantly different at $P < 0.05$.

Table.S2. Thermodynamic parameters of the crystallization and melting processes of CLW, BW, RBW, and CRW oleogels at critical concentration, 6, 8, and 10 wt.% (25 °C).

Sample	Crystallization process				Melting process			
	T_g(℃)	T_{c1}(℃)	T_{c2}(℃)	ΔH_c(J/g)	T_e(℃)	T_{m1}(℃)	T_{m2}(℃)	ΔH_m(J/g)
1%CLW	34.8±0.08^{d}	22.22±0.00^{d}	ND	0.83±0.09^{c}	ND	ND	ND	ND
6%CLW	49.07±0.08^{b}	38.29±0.12^{d}	ND	4.76±0.96^{ab}	64.55±0.58^{c}	48.56±0.02^{d}	ND	5.13±0.29^{a}
8%CLW	51.06±0.04^{bc}	40.40±0.05^{d}	ND	6.55±1.10^{ab}	69.55±0.34^{b}	50.86±0.00^{d}	ND	7.07±0.13^{b}
10%CLW	53.45±0.07^{ab}	43.05±0.02^{d}	ND	8.48±0.84^{ab}	70.22±1.30^{bc}	52.06±0.00^{bc}	ND	9.26±0.66^{a}
2%BW	38.74±0.33^{c}	34.11±0.00^{c}	19.83±0.52^{c}	0.56±0.07^{c}	52.62±0.39^{c}	43.51±0.21^{c}	ND	0.72±0.23^{b}
6%BW	47.71±0.22^{b}	44.34±0.00^{c}	32.04±0.22^{c}	2.83±0.33^{ab}	63.12±0.48^{c}	50.72±0.02^{c}	ND	5.03±0.62^{a}
8%BW	49.39±0.16^{c}	46.17±0.07^{c}	34.81±0.24^{c}	3.84±0.33^{b}	64.49±0.03^{c}	51.74±0.00^{c}	ND	8.82±0.56^{ab}
10%BW	50.56±0.22^{b}	47.52±0.00^{c}	36.02±0.17^{c}	5.00±0.25^{c}	64.17±1.17^{c}	52.86±0.00^{c}	ND	11.40±0.68^{a}
4%RBW	47.26±0.21^{b}	44.99±0.02^{b}	36.34±0.00^{b}	3.52±0.02^{a}	70.75±1.09^{b}	64.64±0.07^{b}	ND	4.70±0.22^{a}
6%RBW	49.47±0.56^{b}	46.68±0.00^{b}	37.04±0.00^{b}	5.32±0.09^{a}	71.69±0.00^{b}	66.09±0.09^{b}	ND	7.23±0.45^{a}
8%RBW	53.02±1.12^{ab}	49.70±0.00^{b}	39.60±0.02^{b}	8.03±0.15^{a}	72.83±0.38^{b}	67.10±0.02^{b}	ND	10.32±0.18^{a}
10%RBW	56.31±1.17^{a}	52.36±0.00^{b}	40.82±0.00^{b}	10.94±0.46^{a}	73.75±0.17^{b}	68.32±0.00^{b}	ND	13.04±0.08^{a}
5%CRW	52.87±0.60^{a}	51.89±0.00^{a}	48.50±0.00^{a}	2.06±0.08^{b}	81.22±0.49^{a}	70.51±0.00^{a}	77.78±0.00^{a}	1.54±0.06^{b}
6%CRW	53.51±0.54^{a}	52.39±0.00^{a}	49.38±0.00^{a}	2.31±0.05^{b}	81.79±0.55^{a}	70.78±0.00^{a}	77.98±0.00^{a}	1.77±0.01^{b}
8%CRW	55.42±0.54^{a}	54.46±0.00^{a}	50.96±0.00^{a}	4.18±0.18^{b}	80.97±1.09^{a}	71.17±0.00^{a}	78.01±0.15^{a}	1.77±0.76^{c}
10%CRW	56.46±0.59^{a}	55.38±0.00^{a}	51.92±0.00^{a}	5.68±0.16^{bc}	81.01±1.17^{a}	71.45±0.00^{a}	77.61±0.44^{a}	2.45±1.27^{b}

Note: The onset temperature (Tg, °C), the first peak temperature (Tc1, °C), and the second peak temperature during crystallization (Tc2, °C); the enthalpy of crystallization (ΔHc, J/g). The first peak temperature (Tm1, °C) and the second peak temperature during melting (Tm2, °C), temperature of complete melting (Te, °C), and enthalpy of melting (ΔHm, J/g). ND stands for non-detect.

References

1. Ogutcu, M.; Yilmaz, E.; Guneser, O. Influence of Storage on Physicochemical and Volatile Features of Enriched and Aromatized Wax Organogels. *J Am Oil Chem Soc* **2015**, *92*, 1429-1443, doi:10.1007/s11746-015-2719-z.
2. Meng, Z.; Qi, K.Y.; Guo, Y.; Wang, Y.; Liu, Y.F. Effects of thickening agents on the formation and properties of edible oleogels based on hydroxypropyl methyl cellulose. *Food Chemistry* **2018**, *246*, 137-149, doi:10.1016/j.foodchem.2017.10.154.
3. Davidovich-Pinhas, M.; Barbut, S.; Marangoni, A.G. The gelation of oil using ethyl cellulose. *Carbohyd Polym* **2015**, *117*, 869-878, doi:10.1016/j.carbpol.2014.10.035.
4. Rosen-Kligvasser, J.; Davidovich-Pinhas, M. The role of hydrogen bonds in TAG derivative-based oleogel structure and properties. *Food Chemistry* **2021**, *334*, doi:ARTN 12758510.1016/j.foodchem.2020.127585.
5. Gao, Y.X.; Lei, Y.J.; Wu, Y.H.; Liang, H.S.; Li, J.; Pei, Y.; Li, Y.; Li, B.; Luo, X.G.; Liu, S.L. Beeswax: A potential self-emulsifying agent for the construction of thermal-sensitive food W/O emulsion. *Food Chem* **2021**, *349*, doi:ARTN 12920310.1016/j.foodchem.2021.129203.
